# Individual and Environmental Factors Associated with Recurrent Falls in Elderly Patients Hospitalized after Falls

**DOI:** 10.3390/ijerph17072441

**Published:** 2020-04-03

**Authors:** Hai Minh Vu, Long Hoang Nguyen, Huong Lan Thi Nguyen, Giang Thu Vu, Cuong Tat Nguyen, Trong Nang Hoang, Tung Hoang Tran, Kiet Tuan Huy Pham, Carl A. Latkin, Bach Xuan Tran, Cyrus S.H. Ho, Roger C.M. Ho

**Affiliations:** 1 Department of Trauma and Orthopaedic, Thai Binh Medical University Hospital, Thai Binh 410000, Vietnam; vuminhhai777@gmail.com; 2 Center of Excellence in Behavioral Medicine, Nguyen Tat Thanh University, Ho Chi Minh City 700000, Vietnam; long.coentt@gmail.com (L.H.N.); pcmrhcm@nus.edu.sg (R.C.M.H.); 3 Institute for Global Health Innovations, Duy Tan University, Da Nang 550000, Vietnam; nguyentatcuong@duytan.edu.vn; 4 Faculty of Nursing, Duy Tan University, Danang 550000, Vietnam; 5 Center of Excellence in Evidence-based Medicine, Nguyen Tat Thanh University, Ho Chi Minh City 700000, Vietnam; giang.coentt@gmail.com; 6 Faculty of Medicine, Duy Tan University, Danang 550000, Vietnam; 7 Department of Ophthalmology, Thai Binh University of Medicine and Pharmacy, Thai Binh 410000, Vietnam; hoangnangtrong@yahoo.com; 8 Institute of Orthopaedic and Trauma Surgery, Vietnam—Germany Hospital, Hanoi 10000, Vietnam; tranhoangtung.vd@gmail.com; 9 Institute for Preventive Medicine and Public Health, Hanoi Medical University, Hanoi 100000, Vietnam; phamhuytuankiet_vkt@fpt.vn (K.T.H.P.); bach.ipmph@gmail.com (B.X.T.); 10 Bloomberg School of Public Health, Johns Hopkins University, Baltimore, MD 21205, USA; carl.latkin@jhu.edu; 11 Department of Psychological Medicine, National University Hospital, Singapore 119074, Singapore; cyrushosh@gmail.com; 12 Department of Psychological Medicine, Yong Loo Lin School of Medicine, National University of Singapore, Singapore 119228, Singapore; 13 Institute for Health Innovation and Technology (iHealthtech), National University of Singapore, Singapore 119077, Singapore

**Keywords:** fall, recurrent fall, older people, environmental factor, Vietnam

## Abstract

Falls and recurrent falls cause great health and social consequences in older people. However, these problems are poorly understood in Vietnam. A cross-sectional study was performed at seven hospitals in Thai Binh province, Vietnam, to investigate the individual and environmental factors associated with recurrent falls among elderly patients hospitalized due to fall injuries in Vietnam. A history of recurrent falls within the last 12 months, sociodemographic, health, and clinical characteristics, as well as environmental conditions, were obtained via self-reported interviews. Multivariate logistic and Poisson regression models were used to identify associated factors. Overall, the mean fall episodes in the last 12 months were 1.8 (Standard deviation—SD = 1.2) episodes, and the 12-month prevalence of recurrent falls was 40.5%. The individual risk factors included not receiving fall prevention guidelines, walking with devices, loss of sensation in hand or foot, and using pain relief medications. The environmental risk factors comprised having too-high stairs and not having dry, clean, and nonslippery bathrooms. This study highlights a significantly high 12-month prevalence of recurrent falls in older patients hospitalized after falls in Vietnam. Moreover, regular assessments of functional disabilities and hazardous environmental conditions, as well as the provision of prevention programs, have potential to prevent falls and recurrent falls.

## 1. Introduction

Falls greatly threaten the health outcomes of elderly people and have become one of the leading health issues in this population [1]. It is estimated that more than 30% of people older than 65 years of age and 50% of individuals older than 80 years old have experienced at least one fall per year [2,3]. Most of the falls caused minor soft tissue injuries, and only 5–10% of the population suffered major problems such as head traumas or fractures [4,5]. However, prior studies underline a vicious cycle where suffering from an initial fall can increase the risk of recurrent falls, which can result in severe complications such as major injuries and frequent hospitalization. In addition, this can lead to the reduction of daily functionality, performance, autonomy, and social independence, as well as elevate the risk of mortality and the burden to caregivers [6,7,8,9,10].

Understanding factors associated with falls plays a vital role in preventing any falls in the future. Worldwide studies indicate that individual factors such as being female, advancing age, experiencing a high number of morbidities, mental problems, cognitive impairment, poor sleep quality, and polypharmacy are related to the increased risk of falls and recurrent falls [11,12,13,14]. Moreover, environmental factors such as poor housing conditions, inadequate lighting, or slippery floors were also considered mediators in precipitating falls [15,16]. Nonetheless, most of the evidence comes from high-income countries. Research on falls in other parts of the world, where 70% of elderly worldwide live, is still lacking [17]. Despite the significant burden of falls, prevention strategies are not prioritized in the policy agendas of governments in low- and middle-income countries [15,16,18]. Therefore, more valid evidence is necessary in each country to design contextualized interventions that eliminate the risk of falls in older people. The objective of this study was to investigate the individual and environmental factors associated with recurrent falls among elderly patients hospitalized due to fall injuries in Vietnam.

## 2. Materials and Methods

### 2.1. Study Design and Participants

This cross-sectional study was performed in seven public hospitals, including one provincial hospital (Thai Binh Provincial General Hospital) and six district hospitals (Kien Xuong, Quynh Phu, Tien Hai, Thai Thuy, Dong Hung, and Hung Ha). They are general hospitals responsible for providing medical care in the entire province and its corresponding districts. These hospitals offer healthcare services in many specialties, such as emergency care, internal medicine, surgery, obstetrics, and gynecology.

Patients who were 60 years of age or older were eligible for recruitment if they were diagnosed with injuries due to falls, used inpatient or outpatient services in selected hospitals, were willing to participate in the study, and gave their informed consent. Patients were excluded if they had any impaired cognition that might influence their capacity to answer the interview or if their caregiver did not allow them to participate. We applied a convenient sampling technique to recruit 430 patients during study periods, and 405 patients agreed to participate (response rate of 94.2%).

### 2.2. Data Collection and Measurement

Data were obtained through face-to-face interviews, which were conducted by well-trained undergraduate medical students at the Thai Binh University of Medicine and Pharmacy. Each interview lasted 15 minutes. Interviewers underwent a two-day intensive training session, which included an introduction to the study, interview techniques, and communication skills. They also participated in a pilot study with ten patients, which ensured they would understand how to collect the highest-quality data consistently. Data of patients in the pilot were not included in the final dataset. The structured questionnaire was developed and piloted the logical order, terms and language in order to assure that patients clearly understood the questionnaire. Next, the questionnaire was revised, and the final version was approved by the principal investigator and the leaders of the hospitals. Eligible patients were determined by the physicians and nurses in each hospital. Once they were chosen for the study, participants received a brief description of the study as well as a description of their rights. If they agreed to participate, they were asked to give their informed written or verbal consent.

Primary outcome: Patients were asked to report the number of fall events they experienced within the last 12 months, including the fall episode that caused their current hospitalization. Recurrent falls or multiple falls were defined if the patient suffered two episodes of falls during this period.

Individual covariates: In this study, we also collected sociodemographic characteristics (age, gender, education, marital status, living arrangements, caregivers, and monthly household income); health and clinical characteristics (number of morbidities, current medications used, polypharmacy use (using more than five drugs); loss of sensation in the hand/foot; history of eye diseases; mobility conditions (physically active/walks with aids/walks with devices); and whether they previously had received guidelines to prevent falls. The six-item Kessler Psychological Distress Scale (K6) was used to assess the psychological distress in older patients hospitalized after falls. This instrument had six items, including nervousness, hopelessness, restlessness/fidgety, depression, “everything was an effort”, and feelings of worthlessness. Each item had five response options from 0 “None” to 4 “Always” [19], resulting in a possible range of a total score from 0 to 24, where the higher score indicated a higher level of psychological distress.

Environmental covariates: We asked patients to report where they lived (urban or rural areas based on the government’s administrative classification and the Law on Urban Planning [20]). They were also asked to describe the conditions of their stair (whether the stair was too high for the patient); floor (whether the floor was dry, clean, flat, and nonslippery (i.e., not wet, smooth, slimy)); bathroom (whether the bathroom was dry, clean, and nonslippery (i.e., not wet, smooth, slimy)); and corridor (whether the corridor was spacious and airy).

### 2.3. Statistical Analysis

A *p*-value of less than 0.5 was considered of statistical significance. Stata software version 15.0 (Stata Corp. LP, College Station, TX, USA) was used to analyze the data. We used the chi-squared test and the Mann–Whitney test to evaluate the difference in the prevalence of recurrent falls regarding the difference of individual and environmental characteristics.

Multivariate logistic and Poisson regressions were performed to examine the possible personal and environmental associated factors with experiencing recurrent falls (Yes/No), and the number of fall episodes in the last 12 months (count variable), respectively. The independent variables included in the full models were age; gender; education; marital status; living arrangements; caregivers; monthly household income; number of morbidities; current medications used; polypharmacy use; loss of sensation in the hand/foot; having a history of eye diseases; mobility conditions; living location; characteristics of stair, floor, bathroom, and corridor. We applied stepwise selection strategies with backward elimination to produce the final regression models. This approach first included all candidate variables, then tested and deleted variables whose loss did not statistically change the model. In this study, we used the threshold of *p*-value of 0.2 as a model fit criterion for this approach, which means that each variable with *p*-value of 0.2 or more was excluded. Only variables that were included were presented in the results.

### 2.4. Ethical Approval

The study protocol was approved by the Institutional Review Board of Thai Binh University of Medicine and Pharmacy (Code: 7641/HDDD).

## 3. Results

During the study duration, 405 patients were recruited. The mean fall episodes in the last 12 months was 1.8 (Standard deviation—SD = 1.2) episodes. The 12-month prevalence of recurrent falls was 40.5%. The rates of recurrent falls were significantly observed regarding living arrangement characteristics (*p* < 0.05). The mean age of older patients in recurrent fall groups was significantly higher (mean = 73.1 years, SD = 8.9, range 60–97 years) than their counterparts (mean = 71.0 years, SD = 9.0, range 60–95 years) (*p* < 0.05). Meanwhile, no difference was found in the prevalence of recurrent falls among gender, education, marital status, and caregiver groups (*p* > 0.05) (Table 1).

Table 2 indicates that there were significant differences in the prevalence of recurrent falls according to the number of morbidities, using pain relief or nonsteroidal, anti-inflammatory drug (NSAID) medications, and mobility status (*p* < 0.05). There were also statistically significant differences between those with/without loss of sensation, history of eye diseases, and those receiving fall prevention guidelines (*p* < 0.05). The mean Kessler-6 score in recurrent fall groups was 4.9 (SD = 2.9), which was significantly higher than those suffering only one fall (mean = 3.5, SD = 2.9) (*p* < 0.05).

Table 3 shows that regarding environmental factors, patients living in rural areas, having too-high stairs, corridors that were not spacious or airy, as well as not having dry, clean, and nonslippery bathrooms were also more likely to have recurrent falls compared to other groups (*p* < 0.05).

Table 4 reveals two regression models. Regarding individual factors, not receiving fall prevention guidelines was associated with recurrent falls in the last 12 months (OR = 2.07; 95% CI = 1.17; 3.66) as well as an increase in the number of fall episodes (Coef. = 0.20; 95% CI = 0.02; 0.37). In the last 12 months, patients who walked with devices had a higher number of fall episodes as compared to those who were physically active (Coef. = 0.22; 95% CI = 0.04; 0.40). Patients who had a loss of sensation in a hand or foot had a 3.59-times-higher risk of recurrent falls compared to those who did not (OR = 3.59; 95% CI = 2.22; 5.80). In addition, older patients using pain relief medications were also 1.85 times more likely to experience recurrent falls in the last 12 months (OR = 1.85; 95% CI = 1.10; 3.12) compared to those not using pain relief medications.

Regarding environmental factors, patients having houses with a high stair height had an increase of 2.54 times the risk of recurrent falls compared to those having stairs with normal height (OR = 2.54; 95% CI = 1.04; 6.19). Similarly, patients without dry, clean, and non -slippery bathrooms were associated with a higher risk of recurrent falls (OR = 2.77; 95% CI = 1.66; 4.62).

## 4. Discussion

Falls and recurrent falls are a significant public health issue in elderly people given their health and social consequences. In this hospital-based study, we found a significantly high proportion of older patients who experienced recurrent falls during the last 12 months. Moreover, we explored some modifiable personal and environmental factors associated with recurrent falls, which could be used to suggest further programs to prevent the occurrence of falls in this population.

In this hospital-based study, we found that 40.5% of older patients suffered from recurrent falls during the last 12 months. This result was higher than a study in the United States (25.0%) [12], comparable to a study in Turkey (45.8%) [11], but lower than a study in the Netherlands (56%) [21]. This diversity is attributable to the differences in study settings and samples. For example, the study in the United States investigated emergency department revisits due to falls in older patients [12], which might underestimate the actual prevalence of recurrent falls because not all fall injuries require hospitalization. Meanwhile, the study in the Netherlands examined falls in patients with dialysis, who might be more vulnerable to recurrent falls compared to our sample [21]. Nonetheless, our findings suggest an alarming issue regarding falls and recurrent falls and an urgent need for appropriate strategies to prevent these problems in older populations.

At the individual level, after adjusting for other covariates, our study found that patients who walked with devices, had a loss of sensation in their hand/foot, or used pain relief medications were more likely to experience recurrent falls. These findings are expected and aligned with previous studies which showed that the rates of falls and recurrent falls were the highest among elderly people with limited functionality or greater disability [22,23,24,25,26]. Moreover, there might be a bidirectional relationship between disability and falls/recurrent falls. In other words, both of these instances can be causes and also consequences [27,28]. Thus, regular examination to detect the functional disabilities as well as performing prompt rehabilitation, such as strength and balance training or tailored physical exercises, are deemed important to prevent falls and recurrent falls in older people.

Notably, the result of the multivariate model showed that patients receiving any fall prevention guidelines were less likely to have recurrent falls. Knowledge and awareness of falls have been shown to be protective factors against falls [29,30]. Several educational interventions indicated positive outcomes in facilitating the awareness of older people in fall prevention, which helps to reduce the recurrent fall incidence in this population [31,32,33]. However, most of our sample had not yet heard of a fall prevention method or did not remember, indicating a gap in knowledge and awareness in older people in the community, especially in the hospital settings. Therefore, education campaigns to improve the knowledge, perception, and practice toward fall prevention are essential to diminish the burden of falls in the older population.

The current study also found an association between environmental factors and recurrent falls. Indeed, hazardous environmental conditions increase the susceptibility of elderly people to falls and recurrent falls [15]. For example, prior studies indicated that footpaths with low quality or unsafe walking areas elevated the risk of falls [34,35]. In our study, too-high stairs or poor bathroom quality were significantly associated with the risk of recurrent falls. These factors should be considered when designing home-based interventions to prevent falls.

This study poses several methodological limitations. First, the nature of the cross-sectional design does not allow us to draw the causal relationships between recurrent falls and associated factors. Therefore, further longitudinal cohorts are warranted to explore the actual relationships. Second, the results of this study come from self-reported information, which could possibly result in recall bias. We addressed this issue by clearly explaining the questions, as well as using probe questions to help patients memorize accurate information. Third, patients were recruited conveniently, which might reduce our ability to generalize about the entire population in other places such as mountainous or remote areas. However, because of the large number of hospitals included in this study, we believe that our findings could be applied in other areas with similar settings. It should be noted that we measured environmental factors by asking patients to subjectively evaluate their stair, floor, bathroom, and corridor conditions. In other studies, age significantly influences the way participants respond to self-reported questions due to the changes of cognitive and communicative functioning, as well as working memory capacity [36,37]. With cognitive decline, older people might face difficulties in recalling the conditions or behaviors mentioned in the questions [38,39], which leads to less accurate answers [40]. We attempted to solve this issue by asking a few additional questions to help them memorize environmental conditions when they fell. Finally, due to limited resources, functional performance measures such as walking speed, balance test, or muscle test were not conducted. Further studies should include these variables in order to provide evidence for more comprehensive interventions to prevent falls among older people in the community.

## 5. Conclusions

This study highlights a significantly high 12-month prevalence of recurrent falls in older patients hospitalized after falls in Vietnam. Findings raise an urgent need for interventions to increase knowledge and awareness of falls and fall prevention. Moreover, regular assessments of functional disabilities and hazardous environmental conditions, as well as the provision of prevention programs, are potential ways to prevent falls and recurrent falls.

## Figures and Tables

**Table 1:** Sociodemographic and behavior characteristics of respondents.

Characteristics	One Fall		Recurrent Falls		Total		*p*-Value
	*n*	%	*n*	%	*n*	%	
**Total**	241	59.5	164	40.5	405	100.0	
**Gender**							
Male	98	40.7	64	39.0	162	40.0	0.74
Female	143	59.3	100	61.0	243	60.0	
**Education**							
<High school	206	85.5	143	87.2	349	86.2	0.62
≥High school	35	14.5	21	12.8	56	13.8	
**Marital status**							
Single	74	30.7	57	34.8	131	32.3	0.39
Having spouse/partner	167	69.3	107	65.2	274	67.7	
**Living arrangements**							
Spouse	141	58.5	94	57.3	235	58.0	0.04
Alone	14	5.8	3	1.8	17	4.2	
Children	64	26.6	58	35.4	122	30.1	
Others	22	9.1	9	5.5	31	7.7	
**Caregiver**							
Spouse	127	52.7	85	51.8	212	52.4	0.05
Children	87	36.1	71	43.3	158	39.0	
Other	27	11.2	8	4.9	35	8.6	
	**Mean**	**SD**	**Mean**	**SD**	**Mean**	**SD**	
**Age**	71.0	9.0	73.1	8.9	71.9	9.0	<0.01
**Household monthly income (thousand Vietnam Dong—VND)**	6383.8	4792.1	6914.3	4798.5	6598.6	4795.9	0.04

**Table 2:** Health and clinical characteristics of respondents.

Characteristics	One Fall		Recurrent Falls		Total		*p*-Value
	*n*	%	*n*	%	*n*	%	
**Number of morbidities**							
0	54	22.4	45	27.4	99	24.4	<0.01
1	106	44.0	54	32.9	160	39.5	
2	64	26.6	38	23.2	102	25.2	
3 or more	17	7.0	27	16.5	44	10.9	
**Current medications used**							
Hypertension drug	75	31.1	46	28.1	121	29.9	0.51
Diabetes drug	11	4.6	6	3.7	17	4.2	0.66
Diuretic drugs	3	1.2	5	3.1	8	2.0	0.20
Pain relief	71	29.5	75	45.7	146	36.1	<0.01
Nonsteroidal, anti-inflammatory drug (NSAID)	21	8.7	40	24.4	61	15.1	<0.01
**Polypharmacy**							
No	237	98.3	159	97.0	396	97.8	0.35
Yes	4	1.7	5	3.0	9	2.2	
**Mobility status**							
Physically active	185	76.8	108	65.9	293	72.4	0.01
Walks with aids	24	10.0	15	9.1	39	9.6	
Walks with devices	32	13.2	41	25.0	73	18.0	
**Loss of sensation in hand/foot**							
No	175	72.6	63	38.4	238	58.8	<0.01
Yes	66	27.4	101	61.6	167	41.2	
**History of eye diseases**							
No	128	53.1	58	35.4	186	45.9	<0.01
Yes	113	46.9	106	64.6	219	54.1	
**Receiving fall prevention guidelines**							
Yes	109	45.2	34	20.7	143	35.3	<0.01
No	94	39.0	97	59.2	191	47.2	
Do not remember	38	15.8	33	20.1	71	17.5	
	**Mean**	**SD**	**Mean**	**SD**	**Mean**	**SD**	
**Kessler-6 score**	3.5	2.9	4.9	2.9	4.1	3.0	<0.01
**Number of days for physical activity per week (days)**	0.4	1.3	0.2	0.9	0.3	1.2	0.51

**Table 3:** Prevalence of recurrent falls according to environmental factors.

Characteristics	One Fall		Recurrent Falls		Total		*p*-Value
	*n*	%	*n*	%	*n*	%	
**Location**							
Urban	26	10.8	6	3.7	32	7.9	<0.01
Rural	215	89.2	158	96.3	373	92.1	
**Height of stair**							
Too high	11	4.6	26	15.9	37	9.1	<0.01
Normal	195	80.9	108	65.9	303	74.8	
Unknown	35	14.5	30	18.2	65	16.1	
**Dry, clean, flat, nonslippery floor**							
Yes	215	89.2	151	92.1	366	90.4	0.34
No	26	10.8	13	7.9	39	9.6	
**Spacious and airy corridor**							
Yes	217	90.0	136	82.9	353	87.2	0.04
No	24	10.0	28	17.1	52	12.8	
**Dry, clean, nonslippery bathroom**							
Yes	188	78.0	83	50.6	271	66.9	<0.01
No	53	22.0	81	49.4	134	33.1	

**Table 4:** Associated factors with recurrent falls and number of fall episodes among elderly.

Characteristics	Recurrent Falls in the Last 12 Months			Number of Fall Episodes in the Last 12 Months		
	OR ^1^	95% CI ^3^	*p*-Value	Coef. ^2^	95% CI	*p*-Value
	INDIVIDUAL FACTORS					
**Marital status**						
Single	REF					
Having spouse/partner	1.43	0.84; 2.44	0.19			
**Living location**						
Urban	REF			REF		
Rural	2.52	0.92; 6.94	0.07	0.25	−0.07; 0.57	0.13
**Monthly household income**	1.00	1.00; 1.00	0.12			
**Number of morbidities**						
0	REF			REF		
1	0.71	0.38; 1.31	0.27			
2	0.54	0.26; 1.09	0.09			
3 or more	1.72	0.67; 4.43	0.26			
**Kessler-6 score**	1.09	1.00; 1.18	0.053	0.02	−0.01; 0.04	0.13
**Receiving fall prevention guidelines**						
Yes	REF			REF		
No	2.07 *	1.17; 3.66	0.01	0.20 *	0.02; 0.37	0.03
Do not remember	2.15 *	1.06; 4.36	0.03	0.14	−0.08; 0.36	0.20
**Mobility condition**						
Physically active				REF		
Walks with aids				−0.03	−0.29; 0.22	0.80
Walks with devices				0.22 *	0.04; 0.40	0.02
**Loss of sensation in hand/foot**						
No	REF			REF		
Yes	3.59 *	2.22; 5.80	<0.01	0.34 *	0.19; 0.49	<0.01
**Using pain relief medication**						
No	REF					
Yes	1.85 *	1.10; 3.12	0.02			
	ENVIRONMENTAL FACTORS					
**Height of stair**						
Normal	REF					
Too high	2.54 *	1.04; 6.19	0.04			
Unknown	1.26	0.66; 2.41	0.48			
**Dry, clean, and nonslippery bathroom**						
Yes	REF			REF		
No	2.77 *	1.66; 4.62	<0.01	0.21 *	0.06; 0.37	<0.01

* *p* < 0.05; ^1^ Odd ratios; ^2^ Coefficient; ^3^ Confidence Interval; REF: Reference group.
